# Predicting Age and Visual-Motor Integration Using Origami Photographs: Deep Learning Study

**DOI:** 10.2196/58421

**Published:** 2025-01-10

**Authors:** Chien-Yu Huang, Yen-Ting Yu, Kuan-Lin Chen, Jenn-Jier Lien, Gong-Hong Lin, Ching-Lin Hsieh

**Affiliations:** 1 School of Occupational Therapy, College of Medicine, National Taiwan University, Taipei, Taiwan; 2 Department of Physical Medicine and Rehabilitation, National Taiwan University Hospital, Taipei, Taiwan; 3 Department of Occupational Therapy, College of Medicine, National Cheng Kung University, Tainan, Taiwan; 4 Institute of Allied Health Sciences, College of Medicine, National Cheng Kung University, Tainan, Taiwan; 5 Department of Physical Medicine and Rehabilitation, National Cheng Kung University Hospital, Tainan, Taiwan; 6 Department of Computer Science and Information Engineering, College of Electrical Engineering and Computer Science, National Cheng Kung University, Tainan, Taiwan; 7 International PhD Program in Gerontology and Long-Term Care, School of Nursing, Taipei Medical University, No. 250 Wu-Xing Street, Taipei, 11031, Taiwan, 886 2-27361661 ext 3614; 8 Department of Occupational Therapy, College of Medical and Health Sciences, Asia University, Taipei, Taiwan

**Keywords:** artificial intelligence, origami, child development screening, child development, visual motor integration, children, developmental status, activity performance, deep learning

## Abstract

**Background:**

Origami is a popular activity among preschool children and can be used by therapists as an evaluation tool to assess children’s development in clinical settings. It is easy to implement, appealing to children, and time-efficient, requiring only simple materials—pieces of paper. Furthermore, the products of origami may reflect children’s ages and their visual-motor integration (VMI) development. However, therapists typically evaluate children’s origami creations based primarily on their personal background knowledge and clinical experience, leading to subjective and descriptive feedback. Consequently, the effectiveness of using origami products to determine children’s age and VMI development lacks empirical support.

**Objective:**

This study had two main aims. First, we sought to apply artificial intelligence (AI) techniques to origami products to predict children’s ages and VMI development, including VMI level (standardized scores) and VMI developmental status (typical, borderline, or delayed). Second, we explored the performance of the AI models using all combinations of photographs taken from different angles.

**Methods:**

A total of 515 children aged 2-6 years were recruited and divided into training and testing groups at a 4:1 ratio. Children created origami dogs, which were photographed from 8 different angles. The Beery–Buktenica Developmental Test of Visual-Motor Integration, 6th Edition, was used to assess the children’s VMI levels and developmental status. Three AI models—ResNet-50, XGBoost, and a multilayer perceptron—were combined sequentially to predict age *z* scores and VMI *z* scores using the training group. The trained models were then tested using the testing group, and the accuracy of the predicted VMI developmental status was also calculated.

**Results:**

The *R*^2^ of the age and the VMI trained models ranged from 0.50 to 0.73 and from 0.50 to 0.66, respectively. The AI models that obtained an *R*^2^>0.70 for the age model and an *R*^2^>0.60 for the VMI model were selected for model testing. Those models were further examined for the accuracy of the VMI developmental status, the correlations, and the mean absolute error (MAE) of both the age and the VMI models. The accuracy of the VMI developmental status was about 71%-76%. The correlations between the final predicted age *z* score and the real age *z* score ranged from 0.84 to 0.85, and the correlations of the final predicted VMI *z* scores to the real *z* scores ranged from 0.77 to 0.81. The MAE of the age models ranged from 0.42 to 0.46 and those of the VMI models ranged from 0.43 to 0.48.

**Conclusion:**

Our findings indicate that AI techniques have a significant potential for predicting children’s development. The insights provided by AI may assist therapists in better interpreting children’s performance in activities.

## Introduction

Origami is a common activity for preschool children [1]. From an occupational therapist’s perspective, the quality of origami products may reflect a child’s developmental age and visual-motor integration (VMI) [2]. For example, a 2-year-old child with typical development is able to roughly fold a sheet of paper in half, and a 3-year-old child is able to fold one diagonally and precisely. Therefore, origami activities and their products could help therapists identify children’s development levels, including developmental age and VMI development, through the activity’s process and the quality of the origami products.

Origami can be used by therapists as an evaluation tool to identify children’s development in clinics because it is easy to implement, attractive to children, requires little time, and uses a simple material (only a piece of paper). However, therapists typically judge children’s origami products based mainly on their own background knowledge and clinical experience, providing subjective and descriptive comments on the products (descriptive information). A few developmental assessments have used origami to assess children’s fine motor skills. For example, the Peabody Developmental Motor Scales, Second Edition [3]; the Comprehensive Developmental Inventory for Infants and Toddlers [4]; the Stanford-Binet Intelligence Scale, Fifth Edition [5]; and the Bruininks-Oseretsky Test of Motor Proficiency, Second Edition [6] all contain one or more items involving paper folding to assess VMI, visual-spatial skills, or fine motor skills. However, these items are relatively simple and cannot solely be used to predict a child’s developmental status. A study by Travers et al [7] investigated how children fold paper across early childhood, and they found that paper folding emerges as early as 27 months and becomes more accurate with age. Moreover, at least 50% of children between 4 and 5.54 years of age completed folds. However, their study only demonstrated children’s ability to fold paper at various ages and provided no information on other developmental statuses. As a result, whether origami products can be used to predict children’s development remains unsupported by evidence.

Artificial intelligence (AI) can be defined as a computer that acts or thinks like a human. Two AI techniques widely used in the medical and rehabilitation fields are computer vision and data prediction [8-10]. These two techniques may provide objective and quantitative data on origami products. Computer vision can transform image features into numerical data for data prediction, whereas the AI technique of data prediction uses data mining and probability to predict or estimate specific outcomes. Compared to traditional statistical analyses, AI data prediction has more flexibility to deal with categorical, ordinal, and numerical data. By combining the techniques of computer vision and data prediction, the important features of origami images can be extracted and further used to predict outcomes of interest for occupational therapists (eg, the children’s age and scores of the standardized VMI assessment in our study).

Some studies have shown that AI techniques can analyze images and can identify children’s behaviors and diagnoses with high accuracy [10-12]. In our preliminary study, we employed a simplified AI model (a single AI model) with a small sample size (n=119) to use photographs of origami products to predict children’s ages [13]. The AI models showed promising performances (*R*^2^=0.10‐0.69). To extend upon our previous works, there were two purposes of our study. First, we refined the AI models, using a combination of 2 types of models, with a larger sample of origami products to predict children’s ages (*z* scores), VMI levels (*z* scores), and VMI developmental status (typical, borderline, or delayed). Second, we explored the performances of the AI models using all combinations of photographs taken from different angles.

## Methods

### Participants and Procedure

The data included in our study were collected as part of an ongoing AI project (An Artificial Intelligence System for Assessing Gross Motor and Fine Motor, Handwriting, Attention and Emotion in Children with Developmental Delay), and some of the data were used in the preliminary study [13]. Preschool children enrolled in kindergarten were recruited to the study. The inclusion criteria were as follows: aged 2-6 years, ability to complete group administrations, and informed consent given by caregivers. The exclusion criterion was any obvious physical or mental disability.

**Figure 1.**
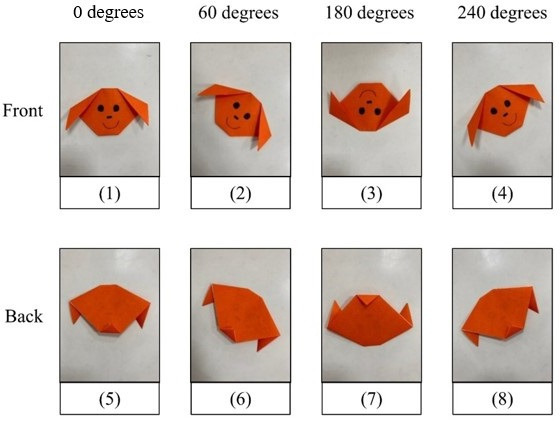
Origami photographs captured from different angles.

The researchers first explained research purposes and procedures to the kindergarten principals. After the kindergarten principals agreed to participate in this study, researchers then contacted the children’s parents to invite them to participate in the study. The researchers visited the children’s classroom at the kindergarten and led them in two group activities (ie, origami and the administration of the VMI subtest). During the group activities, one researcher was a leader, and the other researchers (2-3 people) were coleaders who provided assistance to the children. Each coleader helped 3‐6 children, depending on the number of children in the class. After leading the group activities, the researchers took photographs of the products. The researchers took 8 photographs of each origami work, including 4 angles (0, 60, 180, and 240 degrees) from the front side and the same 4 angles from the back side (Figure 1; the photographs are numbered 1 to 8 for each child depending on the shooting angle).

### Measures

#### Origami

An origami dog was made by each of the children (see example in Figure 1). The dog was constructed in three steps: (1) fold a square paper into a triangle shape; (2) from the 2 corners of the long side of the triangle, diagonally fold 2 small triangles as 2 ears; and (3) turn the paper over, and from the other corner, fold a small triangle as the dog’s chin. After the dog’s face was made, the origami was turned to the front side. Facial features were then drawn on the dog by the child.

#### BEERY VMI Beery-Buktenica Visual-Motor Integration, Sixth Edition

The BEERY VMI Beery-Buktenica Visual-Motor Integration, Sixth Edition (BEERY VMI-6) was used to assess the children’s VMI development [14]. The BEERY VMI-6 consists of three subtests: VMI, visual perception, and motor coordination. Only the VMI subtest was used in this study. The VMI subtest contains 30 pictures. A child needed to copy each picture, and the copy was rated as 0 (incorrect) or 1 (correct). The total score of the VMI test ranged from 0 to 30. The total score of the VMI test was transformed into standard scores based on the VMI norm, and the standard scores were classified into 3 developmental statuses: typical, borderline, or delayed. The total VMI scores (raw VMI scores were referred to as the VMI level in this study) and the developmental status were used for the analyses. The BEERY VMI-6 has good psychometric properties [14].

### Statistical Analysis

**Figure 2.**
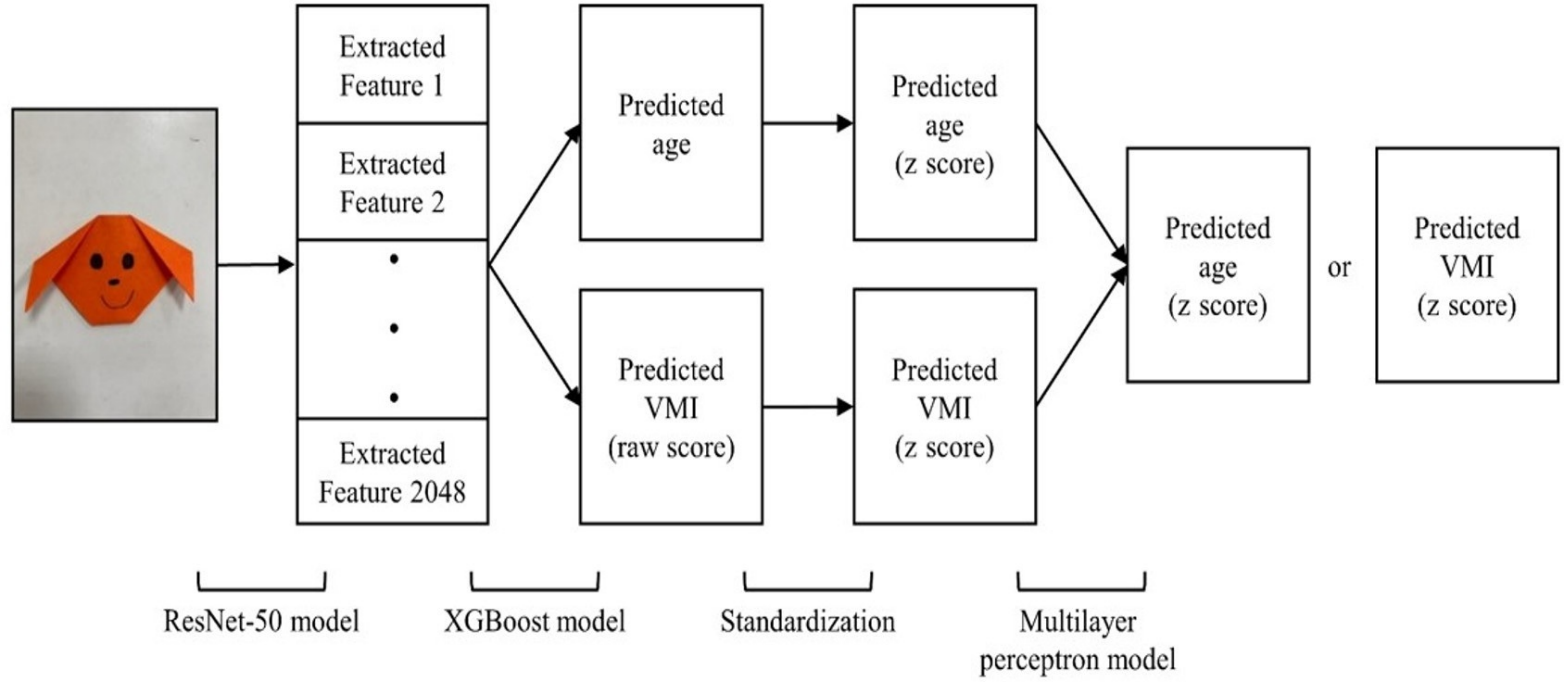
An artificial intelligence model example of the deep learning procedure. VMI: visual-motor integration.

The participants’ products were randomly divided into a training group and a testing group at a 4:1 ratio for the model training and testing. For the training group, 4 steps were conducted to build the AI models that would use origami photographs to respectively predict the children’s age *z* scores and VMI *z* scores (Figure 2). First, we used a deep residual network of 50 layers, which is called ResNet-50 (developed by He et al [15]), to extract origami features of various photos at different angles [16]. Second, we trained XGBoost 1.0.0 models (developed by Chen and Guestrin [17]) to use a child’s photo features to respectively predict the child’s age and raw VMI score. ResNet-50 extracted 2048 features from the photos and the XGBoost model predicted age and raw VMI scores. All combinations of the extracted photo features (from 1 photo alone to 8 photographs together) were respectively fitted to the XGBoost models. The parameters of the XGBoost model were set as follows: n_estimators=100, random_state=1, max_depth=3, and subsample=0.8. The parameters of the multilayer perceptron model were set as follows: random_state=1, max_iter=300, tol=1e^-3^, and n_iter_no_change=5.

Third, the ages and the raw VMI scores predicted by the XGBoost models were respectively transformed into *z* scores (ie, a predicted age *z* score and a predicted VMI *z* score). We adopted the mean and SD of the training group’s age and raw VMI scores for the transformation of *z* scores. We did so because the variables of age and raw VMI score were rated on different scales, and the standardization step ensured that the scales of the age and VMI score would be consistent (both between –1 and 1), which facilitated the subsequent model of data prediction [18,19]. Last, we trained multilayer perceptron models using both the predicted age *z* score and the predicted VMI *z* score to predict the real age *z* score and the real VMI *z* score, respectively.

After the two models (the XGBoost model and the multilayer perceptron model) were trained, the testing group was applied to the two trained models to examine the prediction performance of the models. The *R*^2^, the correlation coefficient, and the mean absolute error (MAE) were used to identify the fitness of the models. The MAE represented the absolute score differences (ie, errors) between the real scores and the predicted scores. It was calculated as follows:


$$\frac{\sum_{i=1}^{n}y_{i}-x_{i}\vee }{n}$$

y_i_ = predicted scores (eg, predicted age and predicted raw VMI scores);

x_i_ = real scores (eg, real age and real VMI scores).

Moreover, we intended to identify if the predicted VMI scores could reflect children’s VMI developmental status. The predicted VMI *z* scores were further used for the analyses. First, the final predicted VMI *z* scores were transformed back to the predicted raw VMI scores. The predicted raw VMI scores were than transformed into predicted standard scores according to the norm of the VMI. Based on the predicted standard scores, we identified children’s VMI developmental status as typical, borderline, or delayed. To examine the accuracy of the AI models in predicting the VMI developmental status, we compared the predicted developmental status to the child’s real developmental status. Python 3.9 (Python Software) was used to perform the model training and the model testing.

### Ethical Considerations

The ethics committee of I-Da hospital approved this study (REC number: EMRP46107N). This study was also approved by the Institutional Review Board of National Taiwan University Hospital (202111093RINB). All methods were conducted in accordance with relevant guidelines and regulations. Informed consent was obtained from the caregivers of the participating children. The study data were deidentified and securely stored in a private cloud space. Participants received a 200 New Taiwan dollar ($6.14 US) coupon after completing the evaluation. No identifying information of individual participants was present in any images included in the manuscript. Participation in this study is entirely voluntary. Participants had the right to withdraw at any time without providing a reason and without facing any negative consequences.

## Results

### Sample Descriptions

A total of 515 children were included in our study. The data for 412 children were used for the training group, and data for the other 102 children were used for the testing group. The sample characteristics and the VMI scores of the children are presented in Table 1. There were no significant differences in age and sex between the training and testing groups.

**Table 1.** Characteristics of the preschool children recruited from kindergartens in the cities of Kaohsiung and Tainan (n=515).

Variables	Training group (n=412)	Testing group (n=103)	*P* value
Age (years), mean (SD)	57.14 (14.46)	57.49 (12.03)	.79
Sex, n (%)			.63
Male	211 (51.2)	50 (48.5)	
Female	201(48.8)	53 (51.5)	
Raw VMI^a^ scores, mean (SD)	11.22 (3.74)	11.34 (4.13)	.78
VMI age-equivalent scores, mean (SD)	58.02 (15.04)	58.70 (16.57)	.99
VMI standard scores, mean (SD)	99.06 (10.32)	99.04 (10.08)	.97
VMI percentiles, mean (SD)	47.51 (22.38)	47.61 (22.77)	.69
VMI developmental status, n (%)			.29
Typical	338 (82)	74 (71.8)	
Borderline	63 (15.3)	27 (26.2)	
Delayed	11 (2.7)	2 (2)	

a VMI: visual-motor integration.

### AI Model Testing

All combinations of photo features were fitted to the XGBoost models, and then the predicted age *z* scores and the predicted VMI *z* scores were fitted to the multilayer perceptron models to produce the final predicted age *z* score and the VMI *z* score. We first inspected the *R*^2^ values of the multilayer perceptron models for predicting the age *z* score and the VMI *z* score to select the better-performing models for further analyses. The *R*^2^ of the age and the VMI models ranged from 0.50 to 0.73 and from 0.50 to 0.66, respectively. We selected AI models that had an *R*^2^>0.70 for the age model and *R*^2^>0.60 for the VMI model, which indicated better performance.

Those models were further examined for their accuracy in predicting the VMI developmental status, the correlations, and the MAE of both the age and the VMI models. Table 2 presents the final performances of the selected models. The accuracy of VMI developmental status was about 71%-76%. The correlations between the final predicted age *z* score and the real age *z* score ranged from 0.84 to 0.86, and the correlations of the final predicted VMI *z* scores to the real VMI *z* scores ranged from 0.7 to 0.81. The MAEs of the age models were between 0.42-0.46, and the MAEs of the VMI models were from 0.43 to 0.48.

**Table 2.** Comparison of origami prediction models using photos with different origami angles.

Photo numbers^a^	*R*^2^ (age)	*R*^2^ (VMI^b^)	Accuracy (VMI)	Correlation (age)	Correlation (VMI)	MAE^c^ (age)	MAE (VMI)
2, 5, 6	0.70	0.61	0.76	0.85	0.79	0.44	0.46
2, 5, 8	0.72	0.66	0.75	0.86	0.81	0.42	0.43
2, 7, 8	0.71	0.61	0.74	0.84	0.78	0.42	0.47
2, 3, 5, 6, 7	0.70	0.63	0.74	0.85	0.79	0.44	0.46
1, 3, 5, 7, 8	0.71	0.60	0.72	0.85	0.78	0.46	0.48
1, 3, 4, 5, 6, 7	0.73	0.61	0.71	0.86	0.78	0.44	0.47
2, 3, 4, 5, 6, 8	0.70	0.62	0.72	0.84	0.79	0.44	0.46
1, 2, 3, 5, 7, 8	0.70	0.62	0.73	0.84	0.79	0.45	0.46
2, 3, 4, 5, 7, 8	0.71	0.63	0.71	0.85	0.79	0.44	0.45
1, 2, 4, 5, 6, 7, 8	0.71	0.60	0.74	0.85	0.78	0.44	0.47

a Numbers correspond to the origami photographs shot from different sides and angles. Please see Figure 1 for the side and angle of each photo.

b VMI: visual-motor integration.

c MAE: mean absolute error.

Among all the combinations of photo features, the combination of photographs 2 (front 60 degrees), 5 (back 0 degrees), and 6 (back 60 degrees) and the combination of photographs 2, 5, and 8 (back 240 degrees) had better performances for predicting children’s age *z* scores and VMI *z* scores.

## Discussion

### Principal Findings

This is the first study to apply AI techniques to origami images for predicting age, VMI level, and VMI developmental status in children. The results showed that AI models could be trained using photographs of children’s origami creations to predict their ages (*R*² ranging from 0.70 to 0.73) and VMI levels (*R*² ranging from 0.60 to 0.66). Furthermore, the accuracy of predicting VMI developmental status was approximately 71%-76%. These findings suggest that AI techniques hold significant potential for analyzing origami images to further assess children’s VMI development.

Analyzing origami photographs using AI techniques to predict children’s age, as done in this study, presents two advantages. First, age is an interval indicator, and an interval indicator is an appropriate outcome measure for testing a prediction model. Second, children’s age, to some extent, reflects their developmental level. As mentioned in the introduction, a 2-year-old child with typical development is supposed to be able to roughly fold a paper in half, and a 3-year-old child should be able to fold a paper diagonally and precisely. Therefore, predicting children’s age through origami may help caregivers and therapists identify children’s developmental status. For example, if an AI model prediction reports that a 5-year-old boy has the origami skills of a 3-year-old, the boy may have a developmental delay in some aspects (eg, fine motor manipulation, visual perception, or VMI). Thus, the boy can be referred for further evaluation and possible intervention. In summary, predicting children’s ages could not only benefit researchers testing the AI model but also provide information about a child’s development for therapists and caregivers.

Comparing all photo combinations for the final models showed that the combinations of photographs 2, 5, and 6 and of photographs 2, 5, and 8 performed better in predicting children’s development. These results indicate that those photo combinations provide more useful information (or important features) about the origami products. This may be because the images of the origami dogs taken from those angles fully capture the dog’s facial features, including the 2 ears, the chin, and the hand-drawn facial features, which could reflect the quality of the origami products. Using a particular and limited number of photographs can promote the efficiency and clinical utility of AI applications.

It is noted that the final predictions of the AI model used the age *z* score and the VMI *z* score as the input and output values. The reason was that the age and the raw VMI scores were two variables based on different scales, and normalization of the two variables could facilitate the model training process [18,19]. Moreover, the predicted age *z* score and the predicted VMI *z* score could be transformed back to the predicted age and predicted raw VMI scores based on the calculation formula. Therefore, future users could obtain the predicted age and the predicted VMI score from the AI model for use in the interpretation of origami performances.

Based on our findings, an evaluation tool for VMI development could be developed and used in both community and clinical settings. This tool would be an app designed to implement an AI model. By allowing users to upload photographs of a child’s origami dogs, the app would immediately calculate and report the child’s predicted age and raw VMI score. This app could provide 4 key benefits. First, the app would offer quantitative data, such as the predicted age, VMI level, and VMI developmental status, derived from a child’s performance in folding origami dogs. Therapists could combine this quantitative information with personal observations to better interpret children’s origami performance. Second, caregivers and teachers, who are not medical professionals, could use the app to better recognize children’s development. The app’s reports could help identify children with delayed VMI development or a predicted age significantly behind their chronological age, prompting caregivers and teachers to refer those children to medical facilities for further evaluation. Third, the app’s internet connectivity would ensure easy accessibility, particularly benefiting children in remote regions or those who cannot undergo standardized evaluations. Fourth, as more people use the app, the continuous input of new data would help refine the AI model, thereby increasing the accuracy of the predicted age, VMI level, and VMI developmental status. This iterative process would enhance the app’s effectiveness over time. Given these advantages, the app has significant potential for application in education and rehabilitation fields. Future research could explore incorporating other origami products into the model prediction to enhance its robustness. Examples include origami airplanes, frogs, and flowers. Allowing children to choose products based on their motivation and preferences could increase the model’s utility and make its predictions of VMI development more robust.

### Limitations

Several limitations were noted in this study. First, a small sample size of children aged 24 to 29 months (n=10) was used. This limited dataset may have resulted in insufficient data for AI learning, potentially increasing prediction errors for this age group. Second, most children recruited in the study exhibited typical development, and those with developmental conditions had mixed diagnoses. Consequently, the results may predominantly reflect the performance of typically developing children. Moreover, the model may tend to predict no developmental issues, increasing the likelihood of false negative results. Third, the images used for the model prediction were restricted to dog origami products, as only these were used to train the model. This limitation restricts the model’s applicability and generalizability to other origami forms. Fourth, due to constraints in evaluation time and research personnel, only the standardized VMI score was used for model prediction. However, executing origami activities also requires manual dexterity, cognition, and visual perception, which were not included in the assessment. Fifth, since the predictions were primarily based on an AI model and conducted via the internet, there are concerns about children’s privacy and data security. Furthermore, it is recommended that clinicians ensure appropriate use and understanding when interpreting the results. Future studies should consider using standardized assessments of other developmental dimensions as model prediction outcomes to provide a more comprehensive understanding of children’s development through origami activities. Additionally, increasing the sample size of young children and children with development conditions and incorporating different origami products in model training would enhance the model’s utility and robustness.

### Conclusion

Overall, it was previously known that origami activities and their products could help therapists identify children’s development levels, including developmental age and VMI development, and photographs of origami could predict children’s age and VMI. However, we have shown that AI techniques can provide quantitative information about activity products, and photographs of origami dogs can be used as an development evaluation tool for children. In conclusion, our study applied AI techniques to images of origami products to predict children’s ages and VMI development. The results demonstrated that children’s ages and VMI development could be predicted using photographs of origami dogs. Given that origami served as a successful example, AI techniques appear to have the significant potential for application to various products of children’s activities, which could provide more quantitative insights.

## Abbreviations

AI: artificial intelligence
BEERY VMI-6: BEERY VMI Beery-Buktenica Visual-Motor Integration, Sixth Edition
MAE: mean absolute error
VMI: visual-motor integration
